# Dynamics of anti-SARS-CoV-2 IgG antibodies post-COVID-19 in a Brazilian Amazon population

**DOI:** 10.1186/s12879-021-06156-x

**Published:** 2021-05-15

**Authors:** Carlos David Araújo Bichara, Ednelza da Silva Graça Amoras, Gergiane Lopes Vaz, Maria Karoliny da Silva Torres, Maria Alice Freitas Queiroz, Isabella Pinheiro Costa do Amaral, Izaura Maria Vieira Cayres Vallinoto, Cléa Nazaré Carneiro Bichara, Antonio Carlos Rosário Vallinoto

**Affiliations:** 1 Laboratory of Virology, Institute of Biological Sciences, Federal University of Pará (UFPA), Belém, Pará Brazil; 2 Amaral Costa Diagnostic Medicine, Belém, Pará Brazil; 3 Graduate Program in Biology of Infectious and Parasitic Agents, Institute of Biological Sciences, Federal University of Pará (UFPA), Belém, Pará Brazil; 4 School of Medicine, Center for Biological and Health Sciences, Pará State University (UEPA), Belém, Pará Brazil

**Keywords:** COVID-19, SARS-CoV-2, IgG, Amazon

## Abstract

**Background:**

In this study, the prevalence and persistence of anti-SARS-CoV-2 (severe acute respiratory syndrome-coronavirus) IgG was evaluated in volunteers 90 days after COVID-19 (coronavirus disease 2019) diagnosis by correlating response dynamics with clinical conditions, epidemiological characteristics, and disease severity.

**Methods:**

The study recruited 200 volunteers aged 18 years or older of both sexes diagnosed with COVID-19. Of the 200 volunteers initially selected, the 135 individuals who underwent serological testing for anti-SARS-CoV-2 antibodies on the first visit to the laboratory, were invited to return, after 90 days, and provide a new blood sample for a second assessment of the presence of anti-SARS-CoV-2 IgG antibody. Disease severity and longevity of symptoms were evaluated for each individual and associated with the serological profile.

**Results:**

Among the 135 individuals who underwent a previous serological test for anti-SARS-CoV-2 antibody, 125 showed reactivity to IgG (92.6%). Of the 125 individuals with detectable IgG in the first test, 87 (69.6%) showed persistence of this antibody after 90 days and 38 (30.4%) lost IgG reactivity in the second evaluation. The frequency of all reported symptoms was higher in individuals who maintained IgG persistence after 90 days of symptoms. Symptom manifestations lasted ≥21 days in the group with a persistent IgG response (39.6%) and ≤ 7 days in the group with a nonpersistent IgG response (50.0%). The length of hospital stay and supplemental oxygen use were higher in individuals with a persistent IgG response.

**Conclusions:**

The results of the present study show a high frequency of loss of anti-SARS-CoV-2 IgG antibodies within 3 months after COVID-19 diagnosis in the Brazilian Amazon.

## Background

The pandemic caused by SARS-CoV-2 (severe acute respiratory syndrome-coronavirus) spread worldwide in early 2020, causing millions of cases and deaths due to COVID-19 (coronavirus disease 2019) [1]. COVID-19 is characterized by several signs and symptoms, including fever, dry cough, dyspnea, headache, chest pain, myalgia, fatigue, nausea, vomiting, diarrhea, abdominal pain, pulmonary infiltrates with fibrosis, and a decreased peripheral lymphocyte count, and may progress to acute respiratory distress syndrome (ARDS) [2].

Several aspects have been investigated to clarify differences regarding the clinical evolution of patients with COVID-19 [3–5]. To date, advanced age and the presence of comorbidities are the main factors associated with disease severity [6]. Immune responses have also been evaluated in terms of both cellular and humoral responses [7–9]. In terms of humoral responses, the production of antibodies against SARS-CoV-2 has been widely investigated by relating their presence to the pathogenesis of COVID-19 or protection against reinfection [10, 11].

Evaluations of the antibody response dynamics for the SARS-CoV species have shown the possibility of variation in the time of IgG seroconversion. Some patients may present late seroconversion of this antibody isotype; that is, seroconversion may occur more than 21 days after the onset of disease symptoms [12]. In this type of infection, IgG levels seem to be related to SARS progression [13].

The antibody-mediated response against SARS-CoV-2 is initially characterized by IgM production, which decreases from the third week, while the IgG response is maintained in patients with COVID-19. In addition, more intense IgM and IgG antibody responses seem to affect patients with severe cases of the disease. These dynamics of the IgG response for SARS-CoV-2 have been shown to be similar among coronavirus species [10].

Although advanced age and the presence of comorbidities are the main risk factors for the development of severe COVID-19, a significant number of individuals do not have these factors but develop severe forms of the disease [14]. Therefore, evaluating the relationships of established risk factors with the effect of the immune response, including the production and dynamics of antibodies, may better elucidate the evolution of COVID-19. Thus, the present study evaluated the prevalence and persistence of IgG in patients in the acute phase of COVID-19 and 90 days after disease diagnosis by correlating these dynamics with clinical conditions, epidemiological characteristics, and COVID-19 severity.

## Methods

### Study population

In this observational cross-sectional study, 200 individuals of both sexes with a previous diagnosis of COVID-19 who attended the Amaral Costa Medicina Diagnóstica Laboratory to perform exams, were selected. The inclusion criteria were age equal to or greater than 18 years, a diagnosis of COVID-19, and residency in the metropolitan region of the city of Belém, the capital of the State of Pará, Brazilian Amazon.

The study participants were selected after advertisement of the research through social media and voluntarily agreed to participate in the study. The volunteers completed a questionnaire designed to collect demographic and social data and information regarding the risk for SARS-CoV-2 infection. Furthermore, clinical and laboratory data were collected. The following tests were considered diagnostic confirmation criteria for COVID-19: IgG-reactive serology, RT-qPCR detection, or chest computed tomography (CT).

Of the 200 volunteers initially selected, the 135 individuals who underwent serological testing for anti-SARS-CoV-2 antibodies on the first visit to the laboratory, were invited to return, after 90 days, and provide a new blood sample for a second assessment of the presence of anti-SARS-CoV-2 IgG antibody.

### Complementary laboratory and imaging tests

Information from the first serological test for anti-SARS-CoV-2 IgG, RT-qPCR molecular biology tests for SARS-CoV-2, and chest CT was obtained and transcribed from the diagnostic reports for each patient. Chest CT was used to assess pulmonary findings suggestive of COVID-19, such as ground-glass opacities and sparse bilateral foci of consolidation, predominantly in the peripheral regions, and the extent of pulmonary involvement was classified as mild (< 25%), moderate (25–50%), or severe (> 50%).

### Sample collection and detection of anti-SARS-COV-2 IgG

Blood samples from all participants were collected in vacuum tubes containing separator gel, and serum was isolated by centrifugation and stored at − 20 °C until anti-SARS-CoV-2 IgG antibody testing, which was performed by qualitative chemiluminescent microparticle immunoassay (CMIA) in an Alinity i automated system (Abbott Laboratories, Chicago, IL, USA) following the manufacturer’s protocol.

### Statistical analysis

All data were stored in a database created in Microsoft Excel, version 2020. Statistical analysis of the data was performed in BioEstat version 5.3. The qualitative data were analyzed using the chi-squared test, Fisher’s exact test, and the G test, and differences were considered statistically significant when the *p*-value was < 0.05.

## Results

The study population consisted mostly of women (65.6%) aged between 19 and 102 years but predominantly younger than 60 years (75.1%). Most of the COVID-19 diagnoses were established by SARS-CoV-2 serological tests (27.0%), followed by chest CT (18.0%) or these two methods combined with RT-PCR (15.3%). More than 95% of the study population consisted of symptomatic individuals with a symptom duration ≥21 days, with only 8% reporting hospitalization and supplemental oxygenation use. The most prevalent blood group was type “O” (44.5%), and most volunteers were immunized against H1N1 (83.0%) and tuberculosis (85.0%).

Among the 200 volunteers in the study, 114 underwent chest CT to assess the extent of pulmonary parenchymal involvement, 65.8% of whom had mild, while 21% had moderate and 6.1% had severe. A normal appearance of the lungs was reported for 7.9% of the volunteers (Table 1).

**Table 1:** Demographic and clinical laboratory data of the study population

	n (%)
**Sex**	
Female	132 (65.6)
Male	68 (34.4)
**Age**	
< 60 years old (mean)	150 (75.1)
≥ 60 years old (mean)	50 (24.9)
**Diagnosis of Covid-19**	
PCR	20 (8.4)
CT	35 (18.0)
Serology	55 (27.0)
PCR + CT + SEROL	29 (15.3)
PCR + CT	14 (6.9)
PCR + SEROL	12 (6.9)
CT + SEROL	35 (17.5)
**Symptoms**	
Yes	197 (98.5)
No	3 (1.5)
**Duration of symptoms**	
≤ 7 days	58 (29.4)
15 days	69 (35.0)
≥ 21 days	70 (35.6)
**Hospitalization**	
Yes	16 (8.0)
No	184 (92.0)
**Supplemental oxygen**	
Yes	16 (8.0)
No	184 (92.0)
**Blood type (ABO)**	
A	55 (27.5)
B	12 (6.0)
AB	5 (2.5)
O	89 (44.5)
Unknown	39 (19.5)
**H1N1 vaccine**	
Yes	166 (83.0)
No	34 (17.0)
**BCG vaccine**	
Yes	170 (85.0)
No	17 (8.5)
Unknown	13 (6.5)
**Pulmonary involvement**	
Normal	9 (7.9)
Mild (< 25%)	75 (65.8)
Moderate (25 to 50%)	24 (21.0)
Severe (> 50%)	6 (5.3)

*PCR* Polymerase chain reaction, *CT* Computed tomography, *SEROL* Serology

One hundred thirty-five volunteers received the SARS-CoV-2 IgG test at the time of COVID-19 symptom presentation, 125 of whom showed SARS-CoV-2 IgG reactivity (92.6%). Among the individuals with SARS-CoV-2-reactive IgG, the most frequently reported symptoms were anosmia, body pain, headache, ageusia, fever, cough, and shortness of breath. In the group without SARS-CoV-2-reactive IgG, the most reported symptoms were fever, sore throat, cough, headache, anosmia, and ageusia. A longer duration of symptoms (≥ 21 days) was observed in the group with SARS-CoV-2-reactive IgG group than in the group without SARS-CoV-2-reactive IgG. However, no statistically significant difference in the frequency of symptoms was found between the two groups evaluated (Table 2).

**Table 2:** The frequency of symptoms in individuals with and without SARS-CoV-2-reactive IgG at the time of diagnosis and the time of symptom onset

Symptoms		1st IgG test		p*
	N	Reactive ***n*** = 125 n (%)	Nonreactive ***n*** = 10 n (%)	
Fever	84	76 (60.8)	7 (70.0)	0.7405
Headache	94	88 (70.4)	6 (60.0)	0.4911
Coryza	62	59 (47.2)	3 (30.0)	0.3422
Cough	85	79 (63.2)	6 (60.0)	0.9994
Sore throat	73	66 (52.8)	7 (70.0)	0.3422
Body pain	94	89 (71.2)	5 (50.0)	0.2818
Abdominal pain	32	32 (25.6)	0 (0.0)	0.1165
Diarrhea	62	58 (46.4)	4 (40.0)	0.7532
Vomiting	16	16 (12.8)	0 (0.0)	0.3681
Nausea	37	37 (29.6)	0 (0.0)	0.0614
Anosmia	95	90 (72.0)	5 (50.0)	0.1640
Ageusia	91	86 (68.8)	5 (50.0)	0.2939
Shortness of breath	60	58 (46.4)	2 (20.0)	0.1843
Hair loss	47	44 (35.2)	3 (30.0)	1.0000
Duration of symptoms				
≤ 7 days	43	40 (32.0)	3 (30.0)	1.0000
15 days	43	39 (31.2)	4 (40.0)	0.7253
≥ 21 days	46	44 (35.2)	2 (20.0)	0.4936
No symptoms	03	2 (1.6)	1 (10.0)	0.2073

*Fisher’s exact test

Of the 125 individuals with detectable IgG in the first test, 69.6% showed sustained detection of this antibody after 90 days; however, in 38 (30.4%) individuals, SARS-CoV-2-reactive IgG was not detected in the second evaluation. Of the 10 individuals without SARS-CoV-2-reactive IgG in the first evaluation, 60% showed seroconversion based on the results from the first test, and 40% remained without detectable SARS-CoV-2-reactive IgG (Table 3).

**Table 3:** The frequency and persistence of SARS-CoV-2-reactive IgG in the study population 90 days after diagnosis

1st IgG test	2nd IgG test		p*
	Reactive (*n* = 93) n (%)	Nonreactive (*n* = 42) n (%)	
Reactive (*n* = 125)	87 (69.6)	38 (30.4)	0.7846
Nonreactive (*n* = 10)	06 (60.0)	04 (40.0)	

*G test

Individuals with SARS-CoV-2-reactive IgG in the second evaluation were considered to have a persistent IgG response, and those without reactive tests were considered to have a nonpersistent IgG response.

Table 4 describes the characteristics of individuals with and without detectable SARS-CoV-reactive IgG 90 days after diagnosis. When comparing the two groups, both predominantly consisted of females (64 and 70.5% with and without a persistent IgG response, respectively) with a mean age younger than 60 years (73.3 and 85.3% with and without a persistent IgG response, respectively). However, the percentage of individuals aged ≥60 years was more frequent in the group with a persistent IgG response than in the group without a persistent IgG response (26.7 and 14.7%, respectively). A predominance of symptomatic individuals was noted in both groups (100 and 94.1% with and without a persistent IgG response, respectively), and symptom manifestations lasted ≥21 days in the group with a persistent IgG response (39.6%) and ≤ 7 days in the group with a nonpersistent IgG response (50.0%). The length of hospital stay and supplemental oxygen use were higher in individuals with a persistent IgG response (10.5%). Regarding vaccination against H1N1 and Bacillus Calmette–Guérin (BCG), no differences were observed between the two groups (88.4 and 79.4% with and without a persistent IgG response, respectively).

**Table 4:** Evaluation of epidemiological and clinical variables according to IgG persistence 90 days after diagnosis

Variables		2nd IgG test		***p***
	N	Persistent ***n*** = 87 n (%)	Nonpersistent ***n*** = 38 n (%)	
**Sex**				
Female	83	56 (64.0)	27 (70.5)	0.6017*
Male	42	31 (36.0)	11 (29.5)	
**Age**				
< 60 years old	94	61 (73.3)	33 (85.3)	0.0773*
≥ 60 years old	31	26 (26.7)	5 (14.7)	
**Symptoms**				
Yes	123	87 (100)	36 (94.1)	0.1902**
No	02	0	2 (5.9)	
**Duration of symptoms**				
≤ 7 days	40	24 (26.7)	16 (50.0)	0.1424*
15 days	39	28 (33.7)	11 (29.4)	
≥ 21 days	44	35 (39.6)	9 (20.6)	
**Hospitalization**				
Yes	11	10 (10.5)	1 (2.9)	0.1756*
No	114	77 (89.5)	37 (97.1)	
**Supplemental oxygen**				
Yes	11	10 (10.5)	1 (2.9)	0.1756**
No	114	77 (89.5)	37 (97.1)	
**H1N1 vaccine**				
Yes	106	71 (81.4)	35 (91.2)	0.1997**
No	19	16 (18.6)	3 (8.8)	
**BCG vaccine**				
Yes	102	71 (88.4)	31 (79.4)	0. 6766**
No	09	5 (5.8)	4 (11.8)	
Unknown	08	5 (5.8)	3 (8.8)	

*Chi-squared test; **G test

The frequency of all reported symptoms was higher in individuals who maintained a persistent IgG response 90 days after diagnosis than in those who presented a nonpersistent IgG response after the same period, but a statistically significant difference was observed only for the cough symptom (*p* = 0.0257) (Table 5).

**Table 5:** Symptoms presented by individuals in the acute phase of COVID-19 according to IgG persistence 90 days after diagnosis

Symptoms		2nd IgG		p*
	N	Persistent ***n*** = 87 n (%)	Nonpersistent ***n*** = 38 n (%)	
Fever	80	60 (69.0)	20 (52.6)	0.1052
Headache	89	63 (72.4)	26 (68.4)	0.6718
Coryza	60	45 (51.7)	15 (39.4)	0.2453
Cough	80	61 (70.1)	18 (47.3)	**0.0257**
Sore throat	66	47 (54.6)	19 (50.0)	0.7011
Body pain	90	66 (75.8)	24 (63.1)	0.1935
Abdominal pain	32	26 (29.8)	6 (15.8)	0.1207
Diarrhea	61	45 (51.7)	16 (42.1)	0.3385
Vomiting	19	14 (16.1)	2 (5.3)	0.1448
Nausea	37	30 (34.4)	7 (18.2)	0.0891
Anosmia	90	66 (75.9)	24 (63.2)	0.3713
Ageusia	86	63 (72.4)	23 (60.5)	0.8447
Shortness of breath	60	46 (52.8)	14 (36.8)	0.1208
Hair loss	47	36 (41.0)	11 (28.9)	0.2303

*Fisher’s exact test

Of the 135 individuals who underwent SARS-CoV-2 IgG tests, only 67 underwent chest CT at the same time for lung assessment. Table 6 shows that all individuals with moderate and severe pulmonary involvement had SARS-CoV-2-reactive IgG and that most of these individuals (19/21) maintained a persistent IgG response after 90 days of infection.

**Table 6:** Radiological parameters of pulmonary involvement according to IgG dynamics at the time of COVID-19 diagnosis and 90 days after diagnosis

Pulmonary involvement		1st IgG test			2nd IgG test		
	N	Reactive n (%)	Nonreactive n (%)	p*	Persistent n (%)	Nonpersistent n (%)	p*
Normal	8	7 (11.9)	1 (12.5)	0.05	3 (5.9)	5 (31.2)	0.02
Mild (< 25%)	38	31 (52.5)	7 (87.5)		29 (56.9)	9 (56.3)	
Moderate/severe (25 to 50%/> 50%)	21	21 (35.6)	0 (0.0)		19 (37.2)	2 (12.5)	

*G test

## Discussion

The population in the present study mostly consisted of women, which differs from the results observed in São Paulo (Brazil) and England where males were more frequent among those diagnosed with COVID-19 [15, 16]. A higher COVID-19 mortality rate in males has also been described in 37 of the 38 countries that provide data by sex [17]. However, the effect of sex on SARS-CoV-2 infection still requires further investigation [18], and genetic, immunological, hormonal, sociobehavioral, economic, and lifestyle factors must be considered to identify such differences [19, 20].

More than 95% of the study population consisted of symptomatic individuals, most likely because individuals with symptoms suggestive of COVID-19 more frequently sought diagnostic laboratory services.

The humoral immune response has been described and suggested as fundamental for protection against SARS-CoV-2 [21, 22]. In addition, the kinetics of the emergence of anti-SARS-CoV-2 antibodies revealed the absence of seroconversion or even a loss of antibodies after infection [23, 24]. Patel et al. [25] reported the 42 and 58% of health professionals from Tennessee (USA) exhibited a persistent IgG response and a nonpersistent IgG response, respectively, 60 days after diagnosis. Robbiani et al. [21] concluded in their study with convalescent COVID-19 patients that many did not have high titers of neutralizing antibodies 39 days after symptom onset. Demonbreun et al. [26] observed loss of anti-SARS-CoV-2 antibody seropositivity after 120 days in 25% of a cohort in Chicago (USA).

The impact of this high percentage of individuals who lost detectable levels of circulating antibodies on the occurrence of SARS-CoV-2 reinfection remains unknown, as has been reported in some countries [27]. Considering that the response time of anti-SARS-CoV-2 antibodies seems to be short in a large portion of the population and that cellular immunity mediated by T lymphocytes is important and lasting [28], Altmann & Boyton [29] suggest that conducting population screening tests for anti-SARS-CoV-2 antibodies and T cells would be very useful.

Most infected individuals generate antibody and T cell responses with magnitudes correlated with the time of infection and disease severity [30]. In this study, more than 95% of the individuals evaluated were symptomatic, with greater IgG persistence among those with symptoms lasting ≥21 days and lower persistence among those with symptoms lasting ≤7 days. The length of hospital stay, supplemental oxygen use, the number of reported symptoms, and moderate and severe pulmonary involvement were also more frequent in individuals with a persistent IgG response, indicating a close relationship between COVID-19 severity and the magnitude of the immune response, as previously suggested [2, 29]. Length of hospital stay and the quality and quantity of the antibody response may be associated with clinical manifestations and disease course, thus justifying further investigations [31].

The percentage of individuals aged ≥60 years was higher in the group with a persistent IgG response. Klein et al. [32] showed that older age was associated with increased antibody responses to COVID-19, which emerged as a factor that can be used to identify individuals with a high probability of presenting strong antiviral antibody responses in contrast to other studies pointing to natural impairment of the immune response in the elderly population due to senescence [33, 34]. However, in addition to senescence, aging is accompanied by changes in the immune profile characterized by chronic subclinical systemic inflammation (inflamm-aging) that may contribute to the disproportionate SARS-CoV-2 mortality rate among elderly patients [35].

## Conclusions

The observed results reveal relationships between female sex, age, symptom duration, and disease severity and the persistence and loss of serum IgG levels in individuals who have recovered from COVID-19 for the first time in the Brazilian Amazon region. The percentage of patients exhibiting antibody loss was high in the present study, which may have implications for seroepidemiological investigations [36], especially those conducted recently, possibly leading to underestimated calculations of the prevalence of infection or even the susceptibility of the population to possible reinfection and thus compromising the success of current vaccination campaigns. Therefore, the use of tests with high sensitivity and specificity as chemiluminescent microparticle immunoassay, can enhance the accuracy and capacity of diagnosis of anti-SARS-CoV-2 antibodies [37].

## Data Availability

Data are available upon request from the corresponding author.

## Abbreviations

SARS-CoV-2: Severe acute respiratory syndrome-coronavirus
COVID-19: Coronavirus disease 2019
SARS: Severe acute respiratory syndrome
ARDS: Acute respiratory distress syndrome
IgG: Immunoglobulin G
IgM: Immunoglobulin M
CT: Computed tomography
RT-qPCR: Reverse transcription real-time polymerase chain reaction
CMIA: Chemiluminescent microparticle immunoassay
ELISAs: Enzyme-linked immunosorbent assays
IL-6: Interleukin-6
IL-1β: Interleukin-1 beta
